# Social Distancing, Health Concerns, and Digitally Empowered Consumption Behavior Under COVID-19: A Study on Livestream Shopping Technology

**DOI:** 10.3389/fpubh.2021.748048

**Published:** 2021-09-16

**Authors:** Qiwei Pang, Haiyang Meng, Mingjie Fang, Jingjing Xing, Jinge Yao

**Affiliations:** ^1^Department of Economics, Sejong University, Seoul, South Korea; ^2^School of Economics and Management, Binzhou University, Binzhou, China; ^3^Department of Logistics, Service and Operations Management, Korea University Business School, Seoul, South Korea; ^4^Department of International Commerce and Business, Konkuk University, Seoul, South Korea

**Keywords:** COVID-19, social distancing, livestream shopping, public health, consumers' behavior

## Abstract

During the COVID-19 pandemic, livestream shopping has provided consumers with a way to maintain social distancing while offering an alternative to offline shopping. This study aims to understand the impact of COVID-19 and other public health crises on the behavioral intentions of consumers using livestream shopping technology. A theoretical model was designed that combines the health belief model, trust theory, and the theory of planned behavior. Empirical data were collected from 358 residents in China and then analyzed using structural equation modeling. The results showed that perceived susceptibility, perceived severity, perceived benefits, and perceived obstacles had a significant impact on consumer trust. Consumer trust in turn had a direct impact on behavioral intention and an indirect impact on behavioral intention *via* attitude. These research results have practical implications for livestream shopping merchants, platform decision-makers, and service designers.

## Introduction

E-commerce has developed rapidly around the world, and it is widely regarded as an indispensable part of the life of consumers. With the continuous development of information and communication technology, livestream shopping based on e-commerce platforms has become a new shopping trend that offers consumers a new shopping experience (1, 2). Livestream shopping technology blends e-commerce technology, social networking technology and entertainment to allow viewers to buy products with just a few taps on their mobile devices. This new form of live streaming has exploded in popularity across East and Southeast Asia, particularly in China (3). In livestream shopping, merchants or streamers can demonstrate or explain their products to consumers in real time, providing their audience with an immersive feeling similar to that associated with offline shopping (4). In addition, when consumers have questions, the seller can answer them immediately. As a result, this innovative combination of e-commerce and livestreaming has experienced explosive growth in its market size (5).

Globally, COVID-19 remains one of the most serious threats to public health despite the recent development and distribution of COVID-19 vaccines (6–8). From May 24 to May 30, 2021, the number of new COVID-19 cases and deaths continued to decrease, with over 3.5 million new cases and 78,000 new deaths reported globally. However, the case and death incidence rates remain at high levels and significant increases have been reported in many countries (9). In this context, the emergence of new livestreaming technology allows consumers to view all of the attributes and details of a product through their mobile devices at home while maintaining social distancing, thus meeting the health needs of consumers (10). The success of livestream shopping is illustrated by the famous Chinese speaker Luo, Yonghao, who attracted 48 million viewers and earned more than RMB 110 million (~US$15.5 million) in his first shopping livestream during the COVID-19 pandemic (2). This new technology has taken on greater importance during the COVID-19 pandemic and other potential public health issues (11).

With the increasing popularity of livestream shopping, research in this area has received significant research attention from various consumer-based perspectives, including consumer loyalty (12), consumer attitudes toward the content of livestream shopping (5), the level of consumer interaction during livestream shopping (13), the intent to use livestream shopping and to purchase products using livestream shopping platforms (2, 14–17). Moreover, public health security incidents such as COVID-19 have been observed to force consumers to practice social distancing (11), which has promoted technological innovations related to livestream shopping. However, a theoretical model that incorporates health crises and livestream shopping-related consumer behavior has yet to be developed.

Therefore, to fill the knowledge gap in this field, the purpose of this study is to develop a targeted research model by determining the reasons and motivations for consumers choosing to use livestream shopping technology during the COVID-19 pandemic. This research is based on the health belief model (HBM) (18) and combines the trust theory (19) and the theory of planned behavior (TPB) (20). The HBM was chosen because it can analyze and identify potential psychological factors associated with the use of livestream shopping technology (7). For example, consumers use this technology as a way to protect themselves from contracting a disease but, when they cannot make face-to-face transactions, the degree of consumer trust in the merchant has a decisive influence on their purchase intentions (21). The theory of planned behavior posits that consumer attitudes will also affect behavioral intentions (22). These theories have been proposed to be appropriate for studying livestream shopping and COVID-19-related topics (23–25).

The remainder of this paper is structured as follows. We present the theoretical foundation of the study and its hypotheses in section Literature Review and Hypothesis Development, while section Methodology describes the data collection and research methodology. Section Results and Discussion presents the data analysis and the results, with section Conclusions summarizing the contributions, limitations, and recommendations for future research.

## Literature Review and Hypothesis Development

### Theoretical Background

**Figure 1:**
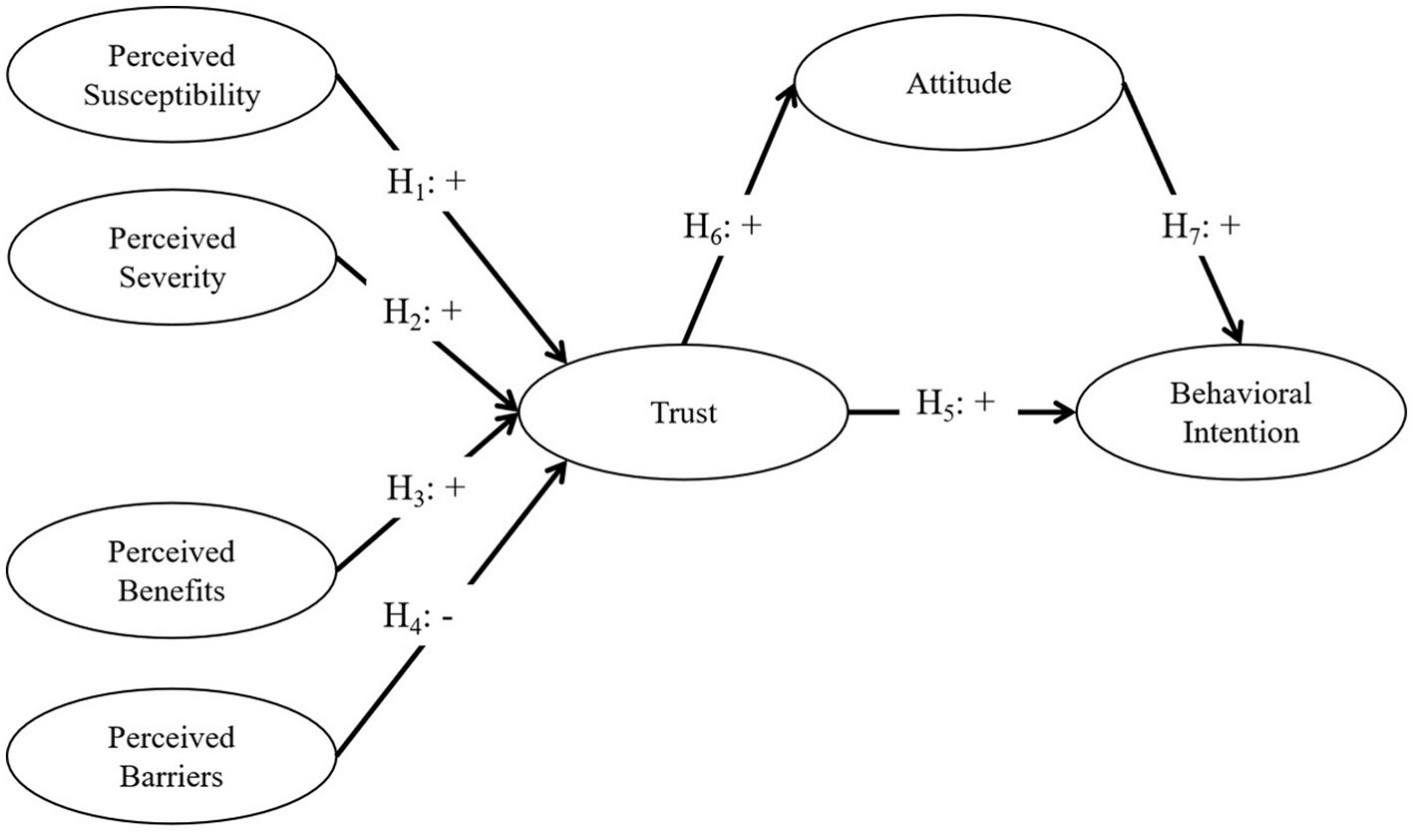
Conceptual framework.

This study is founded on the HBM (18), the trust theory (19), and the TPB (20) (Figure 1). The HBM, developed by Rosenstock and Hochbaum in the 1950s, was the first theoretical model used to explain and predict individual health behaviors (26). It is the most widely used socio-behavioral model for explaining health behaviors and, in particular, for predicting behaviors that avoid a variety of health risks (27). According to the HBM, health decisions are dependent on two factors: risk perception and behavioral evaluation (28). In the context of this investigation, livestream shopping can help consumers avoid being infected by the virus. Risk perception has two sub-components, perceived susceptibility and perceived severity, while behavioral evaluation has two sub-components, perceived benefits and perceived barriers. Examining consumers' health beliefs could provide a more comprehensive understanding of risk perception (29). Therefore, the HBM can be used to logically explain the reasons for consumers' behavioral intentions to use livestream shopping technology.

According to the TPB, behavioral intentions are the immediate antecedent of behavior and can be used as a proxy for assessing actual behavior (22). The TPB is a rational decision-making theory that describes what and how information is processed during deliberative decision-making and proposes that behavior can be best predicted from intentions, which is a measure of how hard people are willing to try and how much effort they intend to put forth in the future (22). The latent constructs of attitude (positive or negative judgment of performing future behavior) and beliefs can predict a consumer's purchase intentions (30). Because both the HBM and TPB feature overlapping dimensions and emphasize the importance of different types of beliefs in customers' purchase intentions, they are frequently employed together (24).

Researchers have frequently added other constructs to improve the explanatory power of the HBM and TPB (31), such as the trust theory. This study defines trust as consumers' confidence in using livestream shopping technology to protect themselves from contracting a disease (7), and proposes trust as a mediator between health belief constructions and customers' intention to use livestream purchasing. The present study proposes trust as a mediator between health belief constructions and customers' propensity to use livestream purchasing. Building client trust is an important component of any business, and this process differs depending on the industry (32). Real-time experience in live chat rooms has replaced text-based human-computer contact since the introduction of live broadcasting. Customers can determine whether or not to buy a product based on the information offered by sellers and member comments, thus it is critical to establish trust (33). Trust is formed when consumers perceive more benefits than risks when using livestream shopping, which can be influenced by the four components of livestream shopping (i.e., perceived susceptibility, perceived severity, perceived benefits, and perceived barriers) (34).

### Hypothesis Development

#### Risk Perception: Perceived Susceptibility and Perceived Severity

In the context of the COVID-19 pandemic, individuals' subjective feelings of personal vulnerability and risk can be understood as perceived susceptibility, whereas perceived severity reveals a person's feelings about the significance of an illness, which can lead to appropriate preventive actions (35, 36). According to Yuen et al. (7), a high perceived safety threat (e.g., perceived susceptibility and perceived severity) indicates a higher likelihood or more severe consequence of infection or potential financial losses, which puts consumers at risk and undermines their trust. Therefore, with online livestream shopping, consumers can reduce the threat of COVID-19 and increase their trust in the platform. Accordingly, based on these research results, we propose the following hypothesis:

**H1: Perceived susceptibility has a positive impact on consumers' trust in livestream shopping**.

**H2: Perceived severity has a positive impact on consumers' trust in livestream shopping**.

#### Behavioral Evaluation: Perceived Benefits and Perceived Barriers

People are willing to devote more time and money to an action when the perceived rewards and related value are larger (37, 38). These benefits would collectively incentivize consumers to use livestream shopping during the COVID-19 pandemic. In this context, perceived barriers would include an individual's perception of the expense and difficulty of taking health precautions (31). Existing research has shown that perceived barriers can significantly reduce consumer trust (39) while, according to Abror et al. (40), perceived benefits is an important antecedent of consumer trust. Chen and Chang (41) also posited that there is a positive association between perceived benefits and consumer trust because a high degree of perceived benefits may boost post-purchase product confidence. In an e-commerce context, the perceived benefits can be defined as a consumer's judgment of the benefits vs. costs when using an online sales platform (42). Therefore, this study proposes the following hypotheses:

**H3: Perceived benefits have a positive impact on consumers' trust in livestream shopping**.

**H4: Perceived barriers have a negative impact on consumers' trust in livestream shopping**.

#### Effect of Trust on Consumers' Behavioral Intention to Use Livestream Shopping

Consumers' willingness to participate in livestream shopping is influenced by their trust in live broadcast platform operators. When a customer has a higher level of trust in the vendor, he/she are more likely to engage in purchasing activity (43, 44). Building online trust is thus critical for vendors to flourish in an e-commerce environment, where transactions are more impersonal and anonymous because it influences consumer behavior and purchasing intent (45). According to Fu et al. (46), customers' perceived utility, contentment, and trust transfer are influenced by external and internal similarity, which influences their purchase behavioral intention. Xu-Priour et al. (47) show that trust and social interaction are positively related to behavioral intention to shop online. Using the unified theory of acceptance and use of technology (UTAUT) as the theoretical framework, Abed (48) insists that social influence and trust are the most significant factors affecting behavioral intention. Therefore, we propose the following hypothesis:

**H5: Trust has a positive impact on consumers' behavioral intention to use livestream shopping**.

#### Effect of Trust on Consumers' Attitudes Toward Livestream Shopping

In the TPB model, attitude is defined as an individual's positive or negative assessment of a behavior based on personal interests, which has a substantial impact on behavior (27, 49, 50). The current study also proposes that trust has an effect on consumer attitudes (51–53). For example, Limbu et al. (51) reported that trust enhances consumer attitudes toward a website and, to encourage consumers to make a purchase, websites must offer superior customer service. Yen (52) indicated that consumers' trust can predict their attitudes of TV shopping services. Furthermore, Lu and Su (53) found that trust can increase consumers' attitude to use mobile shopping. Therefore, it can be assumed that the effect of the HBM constructs on consumer attitude toward livestream shopping is positively mediated by trust.

**H6: Trust has a positive impact on consumers' attitude toward using livestream shopping**.

#### Attitude on Consumers' Behavioral Intention to Use Livestream Shopping

Several recent studies on e-commerce have demonstrated the positive impact of consumer attitudes toward post-pandemic livestream shopping on their purchase intention. On the basis of the TPB, the attitude toward website use, subjective norms, and perceived behavioral control all positively influence online purchasing intentions (54). Wang and Somogyi (55) found that individuals' favorable attitudes toward online shopping positivity influence their behavioral intention toward online shopping. Similarly, Huseynov and Özkan Yildirim (56) showed that positive attitudes toward online shopping play an important role in the formation of online shopping intention for all consumer segments. For these reasons, the following hypothesis is put forward:

**H7: Attitude has a positive impact on consumers' behavioral intention to use livestream shopping**.

## Methodology

### Survey Design and Measurement Items

In line with previous behavioral intention research on livestream shopping, a nationwide anonymous cross-sectional survey was adopted to empirically test the hypotheses (2, 5, 12, 14). The questionnaire consisted of three sections. The first section explained the survey, including the background and purpose, and described livestream shopping technology with examples. The second section asked the respondents for their social-demographic information, including gender, age, education, monthly income, and past experience using livestream shopping. The third section contained a total of 28 items (Table 1) designed to measure seven latent variables: perceived susceptibility, perceived severity, perceived benefits, perceived barriers, trust, attitude, and behavioral intentions. In order to ensure the authenticity of the responses, a question asking the respondents to choose “strongly agree” was added to the third section.

**Table 1:** Scale development.

**Construct**	**Measurement items**	**References**
Perceived susceptibility (PSS)	Strongly disagree (1)/Strongly agree (5)	Yuen et al. (7)
	PSS1. My chance of contracting COVID-19 is low if I use livestream shopping technology.	Huang et al. (31)
	PSS2. Because of my physical health, I am more likely to be infected by COVID-19 if I use offline shopping.	
	PSS3. I most likely will suffer COVID-19 if I use offline shopping.	
	PSS4. I worry a lot about contracting COVID-19 if I use offline shopping.	
Perceived severity (PSV)	Strongly disagree (1)/Strongly agree (5)	Huang et al. (31)
	PSV1. The thought of contracting COVID-19 scares me.	Wang et al. (28)
	PSV2. If I had COVID-19, then my career would be endangered.	
	PSV3. My financial security would be endangered if I had COVID-19.	
	PSV4. If I had COVID-19, my entire life would change.	
Perceived benefits (PBF)	Strongly disagree (1)/Strongly agree (5)	Zhao and Bacao (8)
	PBF1. I experience convenience when using livestream shopping during the COVID-19 pandemic.	Park et al. (57)
	PBF2. I feel that using livestream shopping is safer than traditional shopping during the COVID-19 pandemic.	
	PBF3. Using livestream shopping would be entertaining during the COVID-19 pandemic.	
	PBF4. I feel that using livestream shopping is a beneficial shopping method for people to buy products during the COVID-19 pandemic.	
Perceived barriers (PBR)	Strongly disagree (1)/Strongly agree (5)	
	PBR1. To use livestream shopping, I must give up quite a bit of comfort compared to offline shopping.	Huang et al. (31)
	PBR2. Livestream shopping lacks services and experience	Zhao and An (24)
	PBR3. I don't know how to use livestream shopping to buy products.	Jensen et al. (58)
	PBR4. I think using livestream shopping is a hassle compared to offline shopping.	Wang et al. (59)
Trust (TRU)	Strongly disagree (1) / Strongly agree (5)	Zhao and Bacao (8)
	TRU1. I believe livestream shopping technology is competent and effective in meeting my shopping needs during the COVID-19 pandemic.	
	TRU2. I believe livestream shopping platforms keep customers' interests in mind during the COVID-19 pandemic.	
	TRU3. I believe livestream shopping platforms are trustworthy during the COVID-19 pandemic.	
	TRU4. I believe livestream shopping platforms are honest during the COVID-19 pandemic.	
Attitude (ATT)	Strongly disagree (1) / Strongly agree (5)	Chatzisarantis et al. (60)
	ATT1. I think livestream shopping technology is useful during the COVID-19 pandemic.	
	ATT2. I think using livestream shopping technology is enjoyable during the COVID-19 pandemic.	
	ATT3. I think using livestream shopping technology is interesting during the COVID-19 pandemic.	
	ATT4. I think using livestream shopping technology is beneficial during the COVID-19 pandemic.	
Behavioral intention (BEH)	Strongly disagree (1)/Strongly agree (5)	Ma (2)
	BEH1. I plan to use livestream shopping frequently in the future.	Yuen et al. (7)
	BEH2. My intention is to use livestream shopping when I buy a product next time.	
	BEH3. Livestream shopping will be my preferred mode of shopping in the future	
	BEH4. I would recommend livestream shopping to friends.	

All of the items were developed based on previous research and the opinions of relevant experts and academics to ensure they were valid. Each construct was measured using four items each from the following sources: perceived susceptibility from Yuen et al. (7) and Huang et al. (31); perceived severity from Huang et al. (31); perceived benefits from Zhao and Bacao (8) and Park et al. (57); perceived barriers from Huang et al. (31), Zhao and An (24), Jensen et al. (58), and Wang et al. (59); trust from Zhao and Bacao (8); attitude from Chatzisarantis et al. (60); and behavioral intention from Ma (2) and Yuen et al. (7). A five-point Likert scale ranging from 1 = “strongly disagree” to 5 = “strongly agree” was used to evaluate these items.

To ensure that the survey was appropriate, we conducted a pretest on 16 participants. The respondents reported that they could clearly understand the meaning of the survey after reading the first section. However, two interviewees stated that some of the questions were too long, so some of the questionnaire items were modified to make them more concise.

### Data Collection

This study conducted an anonymous cross-sectional survey in China using Wen Juan Xing, a professional data science company established in 2006. This platform allowed us to conduct representative samples and has been used by many researchers to collect data in cross-sectional studies to investigate people's attitudes (61). The questionnaire was aimed at Chinese residents because the local health risks in China were low and people had become accustomed to implementing safety precautions at the time of the survey, thus raising the need to reassess the respondents' views at this time (7). To ensure the full understanding of each question item, the survey was written in Chinese. It was distributed to a number of WeChat groups related to online shopping, with the respondents told they could receive a digital gift at random upon completion of the survey. The survey was conducted from June 6 to June 25, 2021. A total of 436 responses were collected. After excluding invalid surveys (i.e., the screening question was answered incorrectly or the answering time was too short), 358 valid surveys were used for further analysis. (a conversion rate of 82%).

### Demographic Statistics

As shown in Table 2, the proportion of male and female respondents was 43.6 and 56.4%, respectively. The proportion of female is marginally larger than male. A total of 18 (5%) respondents were 10–19 years old, 180 (50.3%) were 20–29 years old, 74 (20.7%) were 30–39 years old, 56 (15.6%) were 40–49 years old, and 30 (8.4%) were over 50 years old. The majority of respondents are either young or middle-aged. In terms of their education show that they have a high level of education, 195 (54.5%) graduated from a University or college, 33 (9.2%) had studied at graduate school, and 117 (32.7) were studying at University or college at the time of the survey. The monthly average income had the following distribution: 126 respondents earning 3,501–6,000 yuan (35.2%), 135 earning 6,001–10,000 yuan (37.7%), 36 earning 10,001–15,000 yuan (10.1%), and 23 earning over 15,000 yuan (6.4%).

**Table 2:** Respondent demographics.

**Items**	**Category**	**Frequency**	**Percentage (%)**
Gender	Male	156	43.6
	Female	202	56.4
Age (years)	10–19	18	5.0
	20–29	180	50.3
	30–39	74	20.7
	40–49	56	15.6
	>50	30	8.4
Education	High school or below	13	3.6
	Undergraduate	117	32.7
	Bachelor	195	54.5
	Postgraduate or above	33	9.2
Monthly income (yuan) (1 yuan = 0.1549 USD^*^)	<3,500	38	10.6
	3,501–6,000	126	35.2
	6,001–10,000	135	37.7
	10,001–15,000	36	10.1
	>15,000	23	6.4

* *As of July 5, 2021*.

## Results and Discussion

### Measurement Model Assessment

Confirmatory factor analysis was conducted to examine the overall fitness, reliability, and validity of the measurement items. The model fit indices included the chi-square fit index, comparative fit index (CFI), Tucker-Lewis index (TLI), root mean square error of approximation (RMSEA), and standardized root mean square residual (SRMR) (Table 3). Meanwhile, the following criteria [the ratio of chi-square to degrees of freedom (χ^2^*/df*) < 3, comparative fit index (CFI) > 0.9, adjusted Tucker–Lewis index (TLI) > 0.9, and root mean square error of approximation (RMSEA) < 0.08, standardized root mean square residual (SRMR) < 0.08] were applied to evaluate the fitness of the model (62). As the results show, our model fit indices (χ^2^*/df* = 2.17, *p* < 0.05, CFI = 0.96, TLI = 0.95, RMSEA = 0.05, and SRMR = 0.06) all passed their respective minimum cut-off points proposed by Hu and Bentler (62). The results also indicated that all of the criteria satisfied the recommended cut-off values of the measurement model.

**Table 3:** Confirmatory factor analysis results.

**Construct**	**Item**	**Λ**	**AVE**	**CR**
PSS	PSS1	0.830	0.790	0.938
	PSS2	0.906		
	PSS3	0.923		
	PSS4	0.893		
PSV	PSV1	0.785	0.606	0.860
	PSV2	0.796		
	PSV3	0.793		
	PSV4	0.738		
PBF	PBF1	0.809	0.662	0.887
	PBF2	0.868		
	PBF3	0.831		
	PBF4	0.742		
PBR	PBR1	0.877	0.732	0.916
	PBR2	0.901		
	PBR3	0.853		
	PBR4	0.787		
TRU	TRU1	0.762	0.609	0.862
	TRU2	0.770		
	TRU3	0.796		
	TRU4	0.794		
ATT	ATT1	0.826	0.661	0.886
	ATT2	0.840		
	ATT3	0.833		
	ATT4	0.751		
BEH	BEH1	0.835	0.721	0.912
	BEH2	0.865		
	BEH3	0.864		
	BEH4	0.833		

*Model fit indices: χ^2^/df = 2.17 (p < 0.05, df = 325); CFI = 0.96; TLI = 0.95; RMSEA = 0.05; SRMR = 0.06*.

The reliability and validity of the measurement items were then assessed using the standardized loading factor (λ), average variance extracted (AVE), and composite reliability (CR). When the correlation between each measurement item and corresponding construct (i.e., λ) exceeds 0.7, and when CR is >0.8, the model can be considered reliable (63, 64). As presented in Table 3, the CR for each construct was above 0.862, which indicates that the constructs of the measurement model were reliable. In addition, the standardized factor loadings ranged between 0.742 and 0.923, indicating that all of the measurement items were acceptable.

Table 4 summarizes the results for the validity testing. AVE assesses the validity of the assessment items, which were then investigated further using convergent and discriminant analysis. All of the AVEs in Table 4 were larger than 0.5 (65), representing convergent validity. Furthermore, each construct had an AVE that was bigger than the sum of its squared correlations with the other constructs. As a result, the discriminant validity of all of the constructs was confirmed.

**Table 4:** AVE, correlations, and squared correlations of the constructs.

	**PSS**	**PSV**	**PBF**	**PBR**	**TRU**	**ATT**	**BEH**
PSS	**0.790** ^**a**^	0.177^c^	0.310	0.327	0.295	0.307	0.373
PSV	0.421^b^	**0.606**	0.104	0.275	0.167	0.457	0.254
PBF	0.557	0.322	**0.662**	0.248	0.403	0.138	0.320
PBR	−0.572	−0.524	−0.498	**0.732**	0.255	0.282	0.350
TRU	0.543	0.409	0.635	−0.505	**0.609**	0.206	0.293
ATT	0.554	0.676	0.371	−0.531	0.454	**0.661**	0.288
BEH	0.611	0.504	0.566	−0.592	0.541	0.537	**0.721**

a *AVE values are along the main diagonal*;

b *below main diagonal lists the correlations between constructs*;

c *squared correlations between the constructs are above the main diagonal*.

*The numbers in bold are AVE values, which is a critical validity indicator of the model*.

### Structural Model Assessment

**Figure 2:**
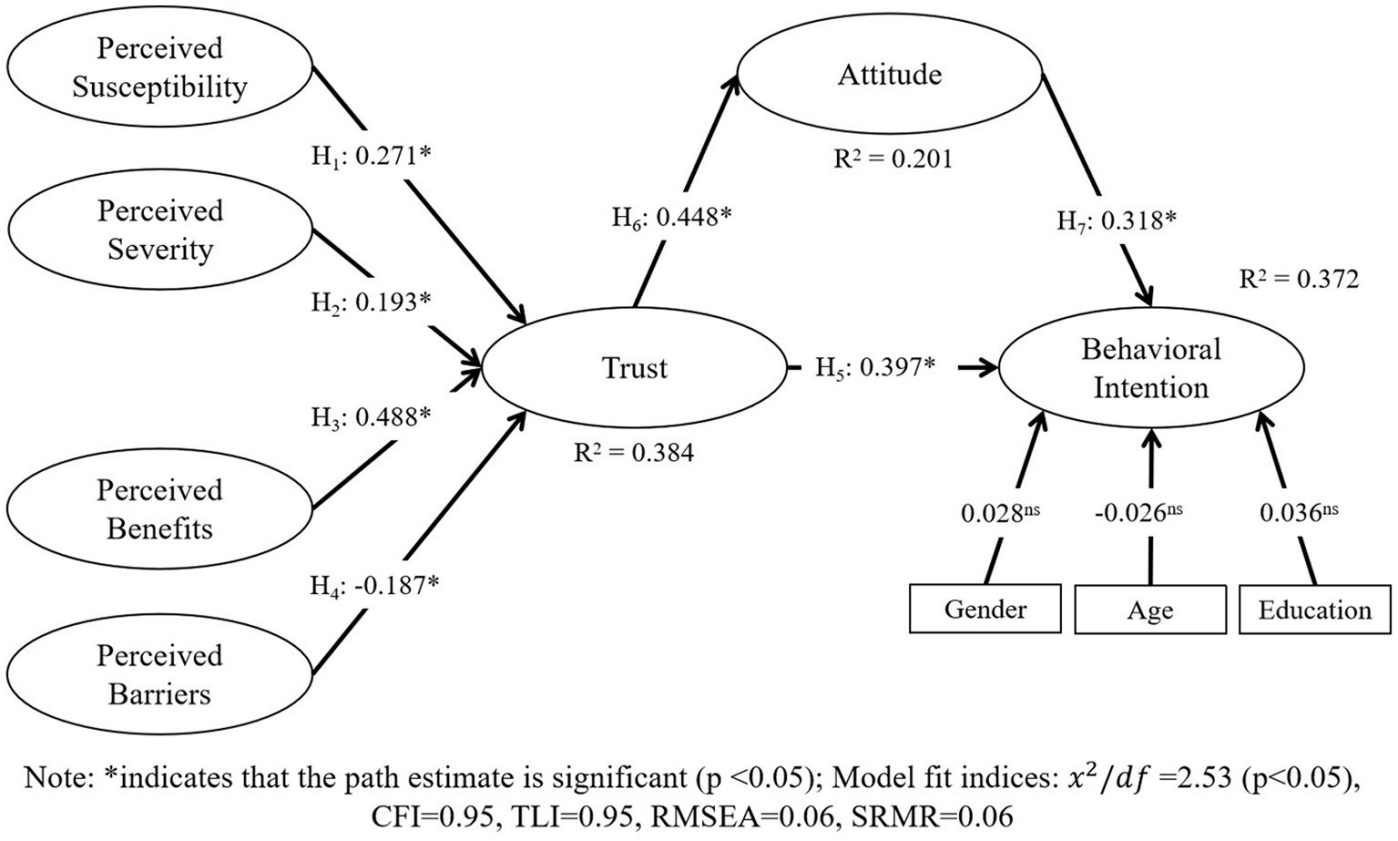
Results of structural model analysis. *indicates that the path estimate is significant (*p* < 0.05); Model fit indices: *x*^2^/*df* = 2.53. (*p* < 0.05), CFI = 0.95, TLI = 0.95, RMSEA = 0.06, SRMR = 0.06.

A structural equation model (SEM) was used in the present study to assess the relationships between the variables. SEM analysis is conducted using AMOS 24. The control variables (gender, age, and education) were included to determine how they affected consumers' behavioral intentions to use livestream shopping technology. The significance and standardized estimated correlations of the constructs were used to test the hypotheses. In addition, the percentage variance explained by the latent variables was determined using squared multiple correlations (*R*^2^). Figure 2 presents the SEM analysis outcomes graphically.

As seen in Figure 2, the model fit of the structural model was sufficient (χ^2^*/df* = 2.53, CFI = 0.95, TLI = 0.95, RMSEA = 0.06, SRMR = 0.06). H1 was accepted because there was a significant positive connection between perceived susceptibility and trust (β = 0.271, *p* < 0.05). H2 was also supported because the positive path from perceived severity to trust was significant (β = 0.193, *p* < 0.05). Perceived benefits and perceived barriers also had a significant impact on trust (*p* < 0.05), with correlations of 0.488 and −0.187, respectively; therefore, H3 and H4 were accepted.

According to the trust theory, consumers trust livestream shopping technology when they sense greater advantages to its use than risks or barriers. During the COVID-19 pandemic, customers' perceptions of the risk associated with using offline shopping instead of livestream shopping technology are reflected in perceived susceptibility and severity within the HBM. That is, when consumers use offline shopping, they will believe that they may suffer from the relevant risk of COVID-19. The greater their perceived susceptibility and severity of COVID-19, the stronger the consumer's trust in livestream shopping. In addition, consumers who express higher perceived benefits believe that livestream shopping offers sufficient benefits to develop trust in it. In other words, they find it more convenient (e.g., experience convenience when using livestream shopping) and fun (e.g., using livestream shopping would be entertaining), while maintaining social distancing and reducing the risk of COVID-19. Finally, higher perceived barriers significantly affect the trust in livestream shopping. In addition to possible flaws in the new technology itself (e.g., poor service and experience), other barriers include personal online shopping habits (e.g., livestream shopping is uncomfortable) and the lack of familiarity with new technologies (e.g., they do not know how to use it). The HBM explained 38.4% of the variance in customer trust (*R*^2^ = 0.384) in this study. Consumers are more likely to trust livestream shopping if they believe the technology offers less risk, more benefits, and fewer restrictions. The results are consistent with previous research (7, 39, 40). However, the result for significant positive effect of perceived susceptibility and perceived severity on trust is contrary to previous studies, such as Yuen et al. (7), who found a significant negative effect of perceived safety threat on consumers' trust about the cruise services. This may because using cruise services is an offline activity, which may lead to a high risk of exposure to COVID-19, while livestream shopping is an online activity without the risk of COVID-19.

Figure 2 shows that consumers' behavioral intention to use livestream shopping was significantly influenced by trust (β = 0.397, *p* < 0.05). As a result, H5 was accepted. This agrees with previous studies (e.g., 44, 61, 62). Even though face-to-face transactions cannot occur when using livestream shopping, if consumers trust the platform and believe that this technology is reliable, then they will be more willing to use this technology. While maintaining social distancing, consumers increasingly seek information about products, brands and merchants, thereby developing their trust in service providers of livestream shopping technology and facilitating their behavioral intention to use livestream shopping technology.

Trust was also found to have a major indirect effect on behavioral intention via attitude. The correlation between trust and attitude was 0.448 (*p* < 0.05), while the correlation between attitude and behavioral intention was 0.318 (*p* < 0.05). As a result, H6 and H7 were supported.

The attitude of consumers represents their evaluation of the benefits, usefulness, happiness, and enjoyment they will feel when using livestream shopping technology. According to the trust theory, when consumers perceive the benefits to be greater than the risk, consumers' trust will be higher. Therefore, trust is required for consumers to form a positive attitude toward the use of livestream shopping technology. In the present study, trust had a direct effect on attitude (*R*^2^ = 0.201), while attitude was positively correlated with consumers' intention to use livestream shopping, which is consistent with the TPB. This suggests that consumers with a positive attitude are more likely to use livestream shopping technology during or after the COVID-19 pandemic. This is in line with previous findings (51–53, 55, 56). For instance, Yen (52) found that consumers' trust can predict their attitudes of TV shopping services.

### Effects Analysis

The impact of the exogenous variables on the endogenous variables was examined in this study (Table 5). In the theoretical model presented in Figure 2, trust fully mediates intention and the four HBM constructs, while attitude partially mediates trust and behavioral intention.

**Table 5:** Direct, indirect, and total effects.

**Exogenous (i)**	**Endogenous (j)**		
	TRU (1)	ATT (2)	BEH (3)
**Direct effect**			
PSS (1)	0.271	-	
PSV (2)	0.193	-	
PBF (3)	0.488	-	
PBR (4)	−0.187	-	
TRU (5)	-	0.448	0.397
ATT (6)	-	-	0.318
**Indirect effect**			
PSS (1)	-	0.121	0.146
PSV (2)	-	0.087	0.104
PBF (3)	-	0.219	0.263
PBR (4)	-	−0.084	−0.101
TRU (5)	-	-	0.143
ATT (6)	-	-	-
**Total effect**			
PSS (1)	0.271	0.121	0.146
PSV (2)	0.193	0.087	0.104
PBF (3)	0.488	0.219	0.263
PBR (4)	−0.187	−0.084	−0.101
TRU (5)	-	0.448	0.540
ATT (6)	-	-	0.318

In terms of the direct impact of the HBM model on trust, the main predictors were perceived benefits (a31 = 0.488), perceived susceptibility (a11 = 0.271), perceived severity (a21 = 0.193), and perceived barriers (a41 = −0.187). Trust was the only direct exogenous variable for attitude (a52 = 0.448) and had a more direct impact on behavioral intention (a53 = 0.397) than did attitude (a63 = 0.318). For indirect effects, perceived susceptibility, perceived severity, perceived benefits, and perceived barriers indirectly affected consumer attitude. As with the direct effects, perceived benefits had the greatest indirect effect on attitude (b32 = 0.219), followed by perceived susceptibility (b12 = 0.121), perceived severity (b22 = 0.087) and perceived barriers (b42 = −0.084). In addition, in descending order, perceived benefits (b33 = 0.263), perceived susceptibility (b13 = 0.146), trust (b53 = 0.143), perceived severity (b23 = 0.104), and perceived barriers (b43 = −0.101) had indirect effects on consumer behavioral intention to use livestream shopping. On this basis, the indirect effects of perceived susceptibility, perceived severity, perceived benefits, and perceived barriers were channeled through a single mediator (i.e., trust) or dual mediators (i.e., trust and attitude).

By adding the direct and indirect effects of attitude on intention, it was found that trust had the greatest total effect on behavioral intention (c53 = 0.540), followed by attitude (c63 = 0.318), which was traced to its direct effect on behavioral intention (a63), perceived benefits (c33 = 0.263). perceived susceptibility (c13 = 0.146), perceived severity (c23 = 0.104), and perceived barriers (c43 = −0.101).

Finally, the control variables (gender, age, and education) were shown to have no statistically significant relationship with and behavioral intention, even though previous studies have shown that the attraction of streamers in livestream shopping varies by gender. When females admired the streamer, they had a more favorable view of the livestream and when males connected with the streamer or other audience members, they generated a more favorable image of the livestream. Furthermore, young people have been shown to prefer livestream shopping because it provides them with a sense of flow, whereas elderly people are unaffected by flow (66). Research has found that consumers with higher professional prestige and college education tend to have stronger behavioral intentions to use new things (67). Regardless, this data supports the theory that theoretical constructs are better predictors of consumer behavioral intention to use livestream shopping than demographic characteristics.

## Conclusions

### Theoretical Contributions

This study makes a number of important contributions to the literature. First, this research enriches the literature on COVID-19 as well as other public health crises and the promotion of online shopping innovation. Due to the widespread impacts of COVID-19, offline shopping cannot meet the needs of consumers who need to maintain social distancing. In contrast, livestream shopping offers consumers this opportunity while also providing an experience similar to offline shopping. In order to understand the impact of the COVID-19 pandemic on the development of online shopping, this study explored the potential factors that influence consumers' use of livestream shopping.

Secondly, based on the HBM, this study combined the trust theory and the TPB to construct a new theoretical model to understand the antecedents of consumers' behavioral intentions to use livestream shopping during public health crises. This theoretical model can be used to explain the psychological impact and the impact of technology itself on consumers during public health crises by exploring their trust and attitudes toward this technology. It was found that higher trust and more positive attitudes toward livestream shopping promote the intention to use this technology. In the context of the COVID-19 pandemic, when consumers perceive that there is a greater chance of COVID-19 infection when shopping offline than with livestream shopping and believe that COVID-19 is a serious matter, their trust in livestream shopping is strengthened. Trust can also be enhanced by increasing the perceived benefits and reducing the perceived barriers. Higher trust also promotes a more positive attitude toward livestream shopping. This is consistent with the results of past studies (7).

Third, this study enriches the trust theory and the TPB. This study evaluated the risks and benefits of using trust as an intermediate factor, while the indirect effect of consumer attitudes on trust and behavioral intention was also discussed. With the full mediation of trust, the four constructs in the HBM had indirect effects on consumers' attitudes and intentions to use livestream shopping. In addition, attitude played a partially mediating role in the relationship between trust and intention. Total effect analysis revealed that consumers' behavioral intention to use livestream shopping was most strongly affected by trust, followed by attitude, perceived benefits, perceived susceptibility, perceived severity, and perceived barriers. Therefore, the mediating role of trust and attitude better reflects the logical reasoning of a consumer leading to their final intention.

### Managerial Implications

This study also offers a number of managerial implications. First, given the significant impact of perceived susceptibility and perceived severity, COVID-19 has had a significant impact on offline merchants and brands. However, livestream shopping can allow consumers to maintain social distancing and enjoy a similar experience to that of offline shopping. Therefore, can ameliorate the negative impact of COVID-19 by offering livestream shopping. Furthermore, service providers merchants and brands that have been providing the services of livestream shopping are encouraged to focus on promoting the safety of using this technology during the COVID-19 pandemic and other potential public health issues.

Second, during the COVID-19 pandemic, the perceived benefits and perceived barriers to livestream shopping had a significant impact on trust. Therefore, service providers that are using or plan to use livestream shopping technology can encourage consumers to use it by increasing the perceived benefits and reducing the perceived barriers. Specifically, they can reduce the perceived barriers by improve the response quality and shorten the response time when consumers use livestream shopping technology to buy products (2). Moreover, they should also pay special attention to provide consumers a pleasant shopping journey rather than pursuing sales to win long-term competitiveness. For instance, the streamers can design some interactive mini games to improve the consumers' perceived benefits.

Thirdly, considering the significant influence and intermediary effect of trust and attitude, livestream shopping platforms and merchants using livestreaming technology should consider publicizing the production and manufacturing process of their products through livestreaming or improving the trust of consumers by optimizing their after-sales service. For example, the disinfection of all goods sold during the COVID-19 pandemic would be desirable. At the same time, publicizing these actions through multiple channels can encourage consumers to form a positive attitude toward the use of livestream shopping technology.

### Limitations and Recommendations

This study has some limitations that should be addressed. First, this study focuses on the impact of COVID-19 on consumers in terms of the behavioral intention to use livestream shopping. Although the benefits and barriers perceived by consumers were investigated, this was limited to the context of the COVID-19 pandemic. Therefore, further research on the determinants of consumers' behavioral intention to use livestream shopping technology in different research settings is encouraged. In addition, this study only studied livestream shopping based on the HBM, the trust theory, and the TPB. It is suggested that further research should apply other relevant theories, such as the spiral of silence theory (68), the opinion leader theory (69), and the uses and gratifications theory (70) from the perspective of communication to study consumers' behavioral intentions in association with livestream shopping platforms. Lastly, the questionnaire of this study was conducted to Chinese residents. However, since the degree of development of livestream shopping technology and consumers' perceptions of the severity of COVID-19 vary from country to country, it is encouraged to conduct relevant research again targeting consumers in other countries.

## Data Availability Statement

The raw data supporting the conclusions of this article will be made available by the authors, without undue reservation.

## Ethics Statement

Ethical review and approval was not required for the study on human participants in accordance with the local legislation and institutional requirements. Written informed consent from the participants' legal guardian/next of kin was not required to participate in this study in accordance with the national legislation and the institutional requirements. Written informed consent was not obtained from the individual(s), nor the minor(s)' legal guardian/next of kin, for the publication of any potentially identifiable images or data included in this article.

## Author Contributions

QP, HM, MF, JX, and JY participated in conceptualization, literature review, data collection, data analysis, and writing of the paper. All authors contributed to the article and approved the submitted version.

## Conflict of Interest

The authors declare that the research was conducted in the absence of any commercial or financial relationships that could be construed as a potential conflict of interest.

## Publisher's Note

All claims expressed in this article are solely those of the authors and do not necessarily represent those of their affiliated organizations, or those of the publisher, the editors and the reviewers. Any product that may be evaluated in this article, or claim that may be made by its manufacturer, is not guaranteed or endorsed by the publisher.
